# Whole-exome sequencing reveals mutational profiles of anorectal and gynecological melanoma

**DOI:** 10.1007/s12032-023-02192-6

**Published:** 2023-10-13

**Authors:** Wei Sun, Kunyan Liu, Hongyu Zhou, Fang Zhao, Yan Dong, Yu Xu, Yunyi Kong, Minghe Wang, Xi Cheng, Yong Chen

**Affiliations:** 1 Department of Musculoskeletal Oncology, Fudan University Shanghai Cancer Center, Shanghai, 200032 People’s Republic of China; 2 Department of Oncology, Shanghai Medical College, Fudan University, Shanghai, China; 3 Department of Bioinformatics, School of Basic Medicine, Chongqing Medical University, Chongqing, China; 4 Department of Gynecological Oncology, Fudan University Shanghai Cancer Center, Shanghai, China; 5 Department of Gynecological Oncology, Minhang Branch of Fudan University Shanghai Cancer Center, Shanghai, China; 6 Department of Dermatology, University Hospital Essen, Essen, Germany; 7 Department of Pathology, Fudan University Shanghai Cancer Center, Shanghai, 200032 China; 8 Department of Colorectal Surgery, Fudan University Shanghai Cancer Center, Shanghai, China

**Keywords:** WES, Anorectal and gynecological melanoma, KMT2D, Tp53

## Abstract

Mucosal melanoma is a rare and highly malignant type of melanoma. Among the sites that mucosal melanoma arises, anorectal and gynecological melanoma has more aggressive behavior and worse prognosis. There was no effective therapy for mucosal melanoma at present. Only a small number of mucosal melanoma patients which harbor mutations in *BRAF* or *KIT* benefit from targeted therapy. So it’s an urgent need to identify more actionable mutations in mucosal melanoma. To identify more potential therapeutic targets in mucosal melanoma, 48 samples were collected from 44 patients with anorectal or gynecological melanoma and subjected to whole-exome sequencing. The tumor mutation burden was low with a median of 1.75 mutations per Mb. In chromosomal level, 1q, 6p and 8q of mucosal melanoma were significantly amplified while 9p, 10p, 10q, 16p and 16q were significantly deleted. Muc16 was the most frequently mutated oncogene in our samples(25%). The mutation frequency of KIT(20%) was comparable to the "triple-wild" genes-NRAS(20%), NF1(20%), and BRAF(11%). KMT2D mutation was found in 18.18% patients, which is previously unidentified. MAPK signaling pathway and lysine degradation were the most frequently mutated pathways. Moreover, patients with TP53 mutations tend to have worse clinical outcome (median survival time 19 vs. 50 months, log-rank *P* = 0.006). 2000 ore mutated genes involved in MAPK signaling pathway were identified, which expand the patients potentially benefit from ample MAPK inhibitors. KMT2D could be a potential therapeutic target. Moreover, *TP53* could be a potential prognosis marker for mucosal melanoma.

**Supplementary Information:**

The online version contains supplementary material available at 10.1007/s12032-023-02192-6.

## Introduction

Melanoma, a highly malignant and lethal neoplasm, arises from pigment-producing cell-melanocyte [1]. Mucosal melanoma (MM) arises from mucosal melanocyte and mainly reported in head and neck regions(31 ~ 55%), anorectum(17 ~ 24%), and female urinary-genital tract(18 ~ 40%) [2, 3]. In fact, MM can arise from any mucosal membrane in the body. MM is a rare subtype and only accounts for ~ 1% of all melanomas in populations with European background [4, 5]. While the incidence of MM in Asian population is higher [6, 7] and has been reported to reach ~ 23% in Chinese people [8, 9]. Despite of its rarity, MM draws great interest due to its aggressive behavior and worse prognosis with 5-year survival ranging from 0 to 20% compared the common counterpart-cutaneous melanoma(CM) [5, 10–13]. Moreover, MM of anorectum and urinary-genital tract has more locoregional nodal metastasis and worse 5-year survival compare to head and neck region [14].

The cause of MM is still unclear. Technologies based on next-generation sequencing fuel our understanding of the genetic basis of cancers. Recently, whole-genome sequencing, targeted sequencing, and whole-exome sequencing is performed on MM to explore the molecular profiles and identify driver mutations [15–22]. In general, it reveals that MM is driven by distinct mechanism from CM. MM has much lower point mutation burden and a higher frequency of somatic copy number alterations and structural variations compare to CM [16–18, 21]. It also reveals that solar ultraviolet radiation (UVR) damage is not a major environmental factor associated with MM [17, 21]. 45 ~ 50% of CM harbors mutations in *BRAF *[17, 23]. However, no single gene reaches such high mutation rate in MM. A few genes are mutated more frequently in MM than in CM, such as *SF3B1*, *KIT,* and *NF1 *[17, 18, 21]. Recently, Yeh’s lab firstly identifies *SPRED1* recurrently amplified in MM and experimentally confers its function as a tumor suppressor particularly in the context of *KIT* mutations [24]. *SPRED1* is also identified as significantly mutated genes in MM by a later study [21]. It is notable that the two studies involve more samples of MMs from “lower” region. It’s likely due to the rarity of samples, the genetic of MM is far from fully explored so far.

The optimal treatment strategy for MM is complete surgical resection of the primary tumor. However, it’s often impossible because the anatomical sites have huge impacts on the quality of patients’ life. Moreover, 50 ~ 90% of patients relapse even achieving negative margins [14]. Adjuvant radiotherapy doesn’t improve the overall survival of this disease though it improved local–regional recurrence [25, 26]. Immunotherapy emerges as a promising strategy to treat melanoma. However, recently clinical trials show that only ~ 23% MM response which much lower compare to CM [27–30]. Unlike CM, only a small number of MM patients which harbor mutations in *BRAF* and *KIT* benefit from targeted therapy. So it’s an urgent need to identify more actionable mutations in MM.

In the present study, 48 primary/metastasis tissue samples from 44 patients with MM of anorectal and gynecological origin were collected and subjected to whole-exome sequencing (WES). Mutation profiles of MMs were explored to identify actionable mutations which pave the way for the exploration of the molecular mechanism of MM.

## Methods

### Patients and samples

Patients are recruited after diagnosed as mucosal melanoma at the Fudan University Shanghai Cancer Center. Written informed consent was obtained for each patient. A total of 48 tumor samples collected from 44 patients from 2013 to 2018, including 24 fresh-frozen tumor tissues from anorectum and 24 formalin-fixed, paraffin-embedded (FFPE) tumor tissues from female urinary-genital tract. The matched peripheral blood sample also collected as normal control tissue for each patient. Notably, there are 2 patients from which primary and local recurrent tumor were both collected. 1 patient from which primary tumor and lymph node metastasis were both collected and 1 patient whose primary tumor and urinary tract metastasis were both collected. The other patients only had primary tumors. All samples were independently reviewed by expert melanoma pathologists prior to inclusion into the study. Patients were long-time followed up. The median follow-up duration was 24 months (range 0–93, with 35 (73%) patients alive and censored at most recent follow-up date). The clinical information and survival data of all patients are list in Table S1. The study was approved by the Ethics Committee of Fudan University Shanghai Cancer Center (FUSCC) and conducted according to the Declaration of Helsinki. Study data were anonymized to protect subjects’ identities.

### Whole-exome sequencing

Genomic DNA was extracted from fresh-frozen tumor tissues, FFPE tissues and peripheral blood using UniversalGen DNA Kit (ComWin, Beijing, China), NuClean FFPE DNA Kit (ComWin, Beijing, China) and Magbead Blood DNA Kit (ComWin, Beijing, China) according to the manufacture’s instruction, respectively. Sequencing library was constructed using DNALibrary Prep Kit (MyGenostics Inc, Chongqing,China). In brief, DNA was fragmented, end repair and adding A to the end. After adapter was added to the DNA fragments, PCR was performed and the PCR products were purified. Exome-enriched fragments were captured by GeneCap WholeExome Enrichment Kit (MyGenostics Inc, Chongqing, China) according to the manufacture’s instruction. Library quality was assessed by Agilent 2100 Bioanalyzer system. Qualified library was sequenced by Illumina Nextseq 500 to generate paired-end 150bp reads.

### Somatic single-nucleotide variation/InDel analysis

Raw reads were trimmed by Trim Galore(v0.4.5) to filter adapter and low-quality reads. Clean reads were aligned to human reference genome (hs37d5) by BWA(v0.7.16a) [31]. Duplications were marked by Picard tools (v2.5.0, http://broadinstitute.github.io/picard/). Somatic single-nucleotide variations (SNV) and small insertions and deletions (InDels) were called by GATK Mutect2(v4.1.2.0) followed by the instruction of best practice suggested by the Broad Institute [32]. Specifically, Mutect2 was firstly used to get the raw variants. Then Learn Read Orientation Model was used to model the sequence context-dependent artifacts account for FFPE deamination. Calculate Contamination was used to measure cross-sample contamination. Raw variants then filtered for the orientation bias artifacts and cross-sample contamination. SNVs then were filtered as follows: (1) sequencing depth ≥ 10X in both tumors and their matched normal samples; (2) ≥ 4 reads to support mutated allele in tumors; (3) a variant allelic fraction in tumors > 5%; (4) the frequency of variants in gnomAD47 is less than 0.01; (5) the frequency of variants are not more than 0.1% in any of the three databases: dbSNP142, the 1000 Genomes Project and EXAC. Putative variants were manually checked using the Integrative Genomics Viewer (IGV) [33]. Final high quality variants were annotated using ANNOVAR (v1.2) for consequence prediction [34]. Tumor mutation burden (TMB) was defined as the number of somatic nonsynonymous SNVs/InDels per million base pair and it was calculated as (the total number of somatic nonsynonymous mutations)/ (the total number of megabases sequenced).

### Significantly mutated genes

MutSigCV(1.41) [35] and OncodriveFML(v2.2) [36] were used to identify significantly mutated genes with a threshold *q* < 0.1 among all high quality variants. MutSigCV and OncodriveFML identified tumor driver genes based on mutation frequency and functional impact of variants, respectively.

### Copy number variation analysis

All somatic copy number variant (CNV) were called by CNVkit (v0.8.3) [37]. CNVkit estimated somatic copy number based on read count method and was designed specifically for data derived from targeted sequencing. Baited regions plus upstream/downstream 50 bp were used as targeted regions. Reads in target regions and unspecifically captured off-target regions were counted within each bin. All matched normal samples were pooled as a copy number reference. Read counts were normalized by GC content, repetitive sequences, target footprint size and spacing from pooled reference and copy number ratio were determined. Copy number segmentation was performed using CBS method. Tumor purity and ploidy were estimated by Sequenza(v3.0.0) [38]. Absolute integer copy number was called with –m clonal and estimated tumor purity and ploidy. Significantly recurrent copy number alteration region was determined by GISTIC2.0 [39].

### KEGG pathway analysis

Genes with recurrently nonsynonymous somatic mutations were overlaid onto pathways defined by the Kyoto Encyclopedia of Genes and Genomes (KEGG) using R package clusterProfiler. A pathway was considered altered in a given sample if at least one gene in the pathway contained a somatic mutation.

### Survival analysis

Kaplan–Meier survival analysis was performed on patients with long-term follow-up information (*n* = 35) based on TMB, the mutational status of all somatic mutated genes, gender, primary sites of tumor and tumor size using R package maftools.

## Results

### Patient and clinical information

Patients were enrolled in this study after diagnosed as mucosal melanoma (MM) at the Fudan University Shanghai Cancer Center. None of them were treated with chemotherapy or radiotherapy before operation. 24 fresh-frozen tumor from anorectum and 24 FFPE tumors from female urinary-genital tract were collected. The matched peripheral blood samples were also collected as normal control tissue for each patient. Overall survival was measured from the date of first operation to time of death or most recent follow-up date (5/30/2018).

### Genomic mutation spectra in MM

**Fig. 1:**
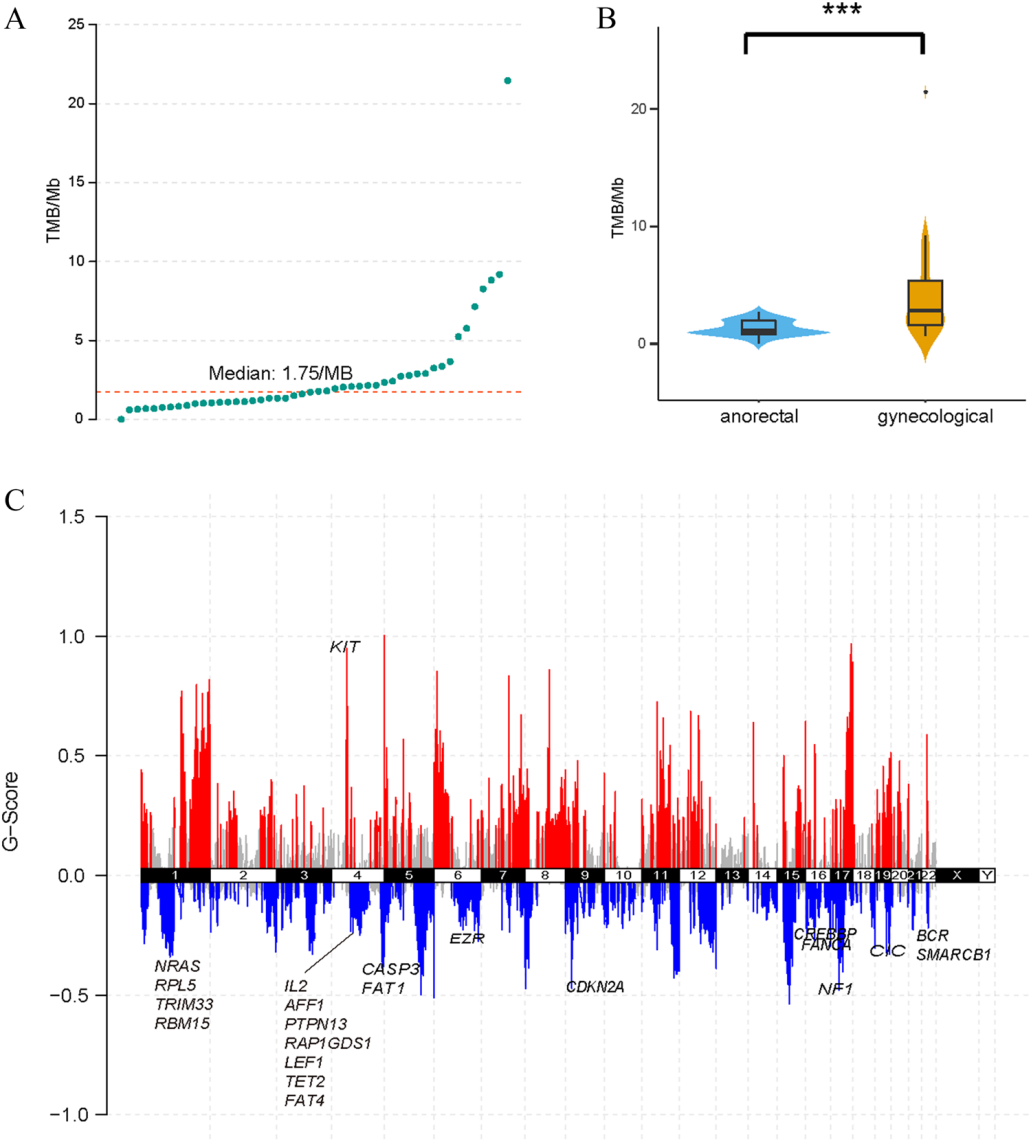
XXXX

We performed whole-exome sequencing on collected samples. On average, ~ 195 Mb reads were generated for each sample. ~ 91.4% of the targeted regions were covered by at least 10 reads. Samples were sequenced to an average read depth of 114x (range 39–206) in the tumor sample and 139x (range 40–268) in the matched normal sample. As reported before [16, 17, 21, 24], MM’s tumor mutation burden (TMB) was low with a median of 1.75 mutations per Mb (range 0–21.44) (Fig. 1A). Tumor from anorectal locations had a significantly lower TMB than which from urinary-genital tracts (Wilcox test, *P* = 7.838e-05) (Fig. 1B). Patients were stratified as high TMB group and low TMB group by percentile. However, it revealed that TMB is not associated with prognosis in our samples (log-rank *P* = 0.66; Figure S1).

Regions that recurrently altered in copy numbers among all samples were identified by GISTIC2. In broad regions (chromosomal arm level), 1q, 6p and 8q were significantly amplified (*q* = 1.39e-07, 0.00924 and 4.8e-05, respectively). 9p, 10p, 10q, 16p, and 16q were significantly deleted (*q* = 0.00942, 1.98e-05, 2.35e-08, 0.00898, and 0.00898, respectively) (Figure S2). Among the genes identified as significantly amplified or deleted by GISTIC2 (*q* < 0.1), we focused on the genes that annotated as known cancer driver genes by COSMIC Cancer Gene Census or reported as melanoma driver genes previously. *KIT* was significantly amplified. *CDKN2A*, *NF1*, *NRAS*, *RPL5*, *TRIM33*, *RBM15*, *CASP3*, *FAT1*, *FANCA*, *EZR*, *CREBBP*, *BCR*, *SMARCB1*, *CIC*, *IL2*, *AFF1*, *PTPN13*, *RAP1GDS1*, *LEF1*, *TET2,* and *FAT4* were significantly deleted (Fig. 1C).

### Recurrent *KMT2D* mutations in MM

**Fig. 2:**
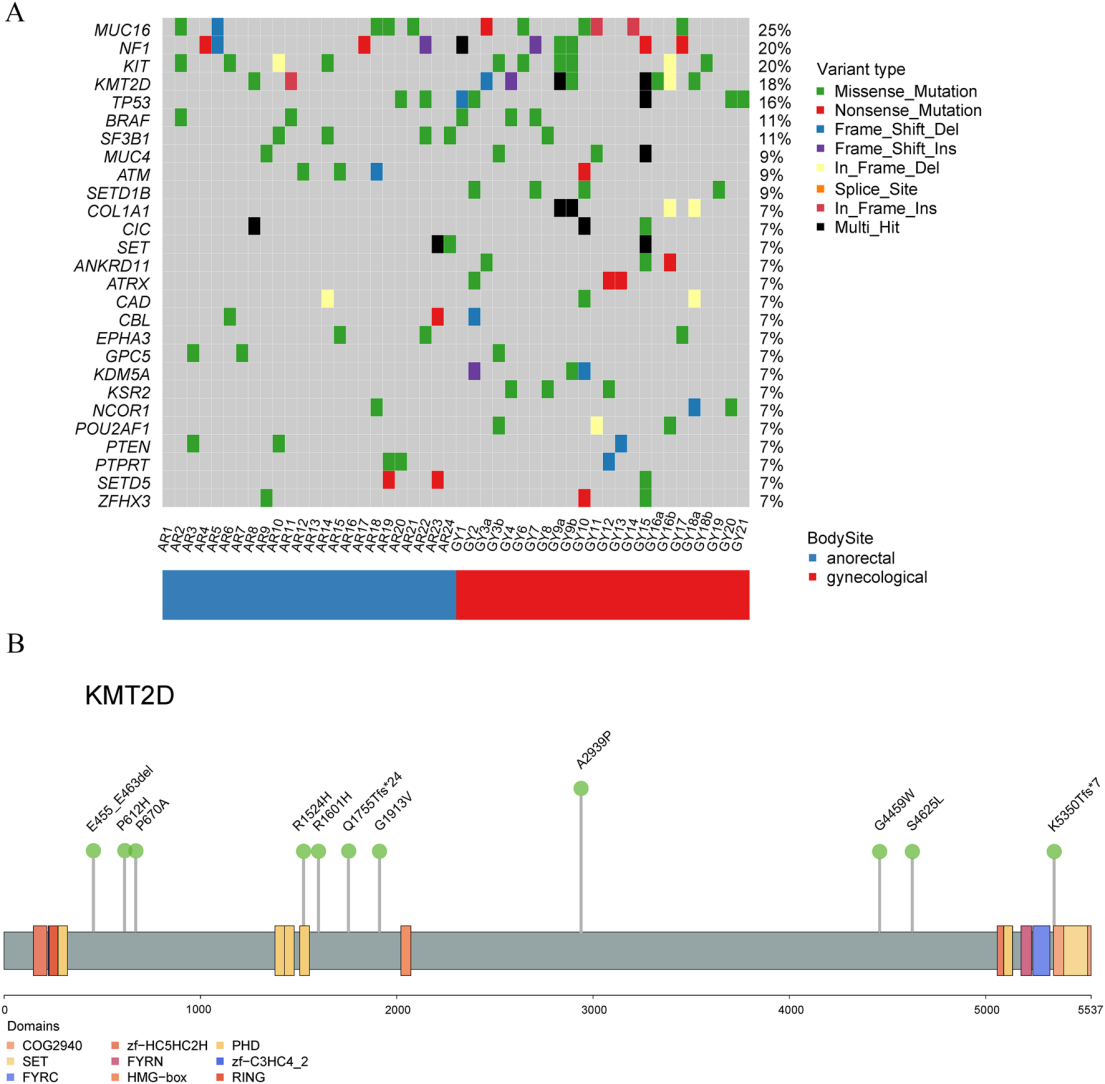
XXXX

*KMT2D*, which encode a histone methyltransferase, was mutated in 18.18% (8/44) patients among which 2 were anorectal and 6 were gynecological (Fig. 2). Increasing evidences shed light on the roles of histone modification proteins in cancer progression [40, 41]. And *KMT2D* was reported as a potential tumor suppressor in other type of cancers [41]. 2 patients had 2 mutation sites in this gene while the other 6 patients had only one mutation. No mutation hotspot was found in this gene. Mutation NM_003482:exon34:c.G8815C:p.A2939P occurred in the primary and metastatic tumors of one patient. One mutations (NM_003482:exon16:c.G4571A:p.R1524H) occurred in the SET domains which was responsible for its histone H3 lysine 4 (H3K4) methyltransferase activity. Notably, *COL1A1* and *GRM3* were significantly co-occured with *KMT2D* (*P* = 0.001 and 0.0069, respectively). Whether the mutational status of *KMT2D* had impact on the clinical outcome was tested. However, there was no significant difference between wild and mutant *KMT2D*.

### Other cancer driver genes in MM

MutSigCV and OncodriveFML were used to find the significantly mutated genes (SMGs) in our samples. Only *NF1* that two software both identified as SMG. *NF1* mutation was reported as a major subgroup of cutaneous melanoma by TCGA. *NF1* had nonsynonymous SNVs/InDels in 4 anorectal and 5 gynecological patients among which primary and metastatic samples of one patient both had mutations and one patient had 2 mutations. Taken together, 20.45% (9/44) of patients were affected by *NF1* mutations. Then, the mutation status of the known cancer driver genes was checked. Genes mutated in at least 3 patients were shown in Fig. 2. *MUC16* had the most frequent SNVs/InDels mutation load with frequency of 25% (11/44) followed by *NF1* and *KIT*. Anorectal samples had less mutated genes than gynecological. *SETD1B, COL1A1*, *ANKRD11*, *ATRX*, *KDM5A*, *KSR2,* and *POU2AF1* mutated only in gynecological samples.

### Recurrently mutated pathways in MM

**Fig. 3:**
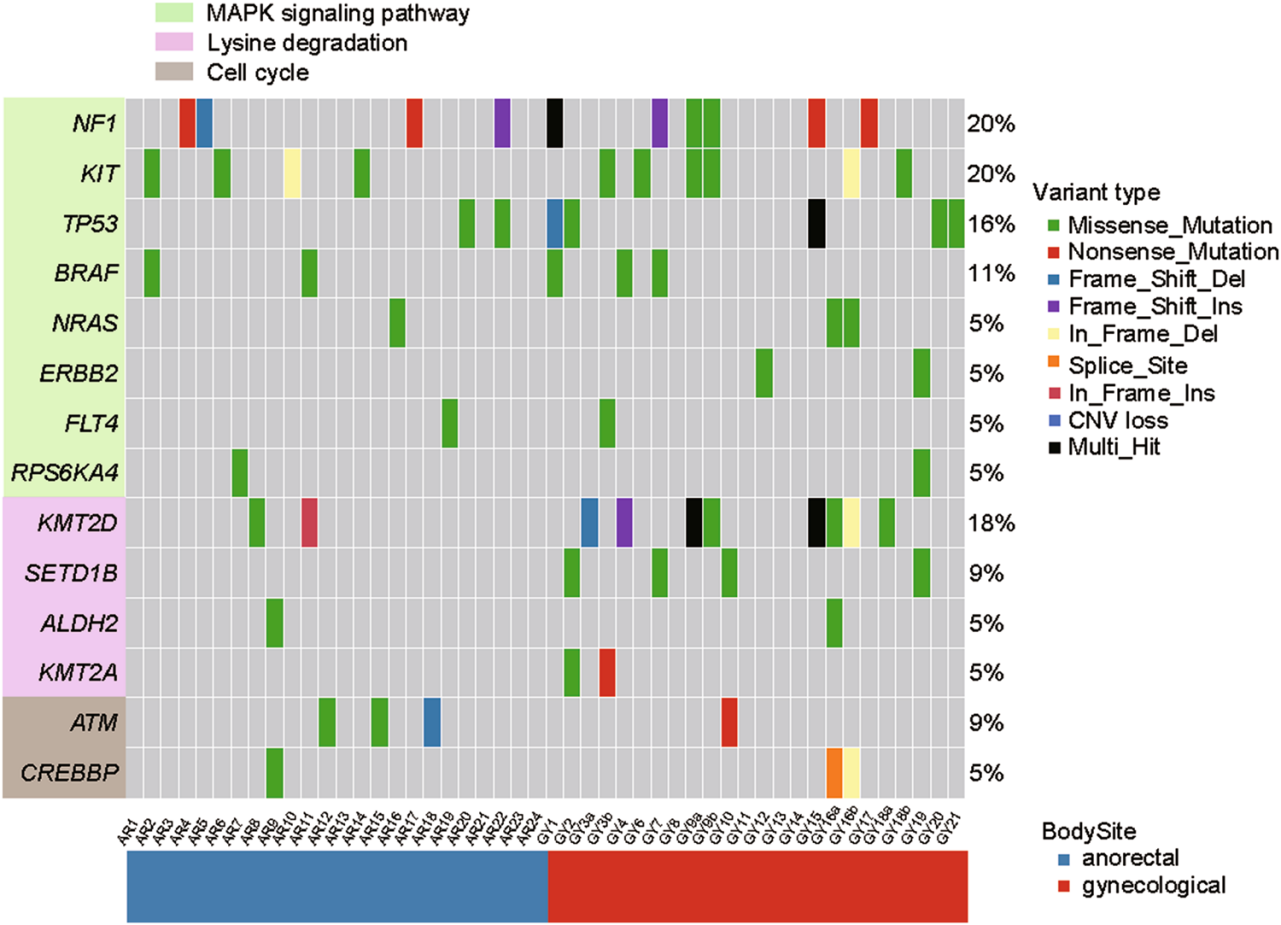
XXX

**Fig. 4:**
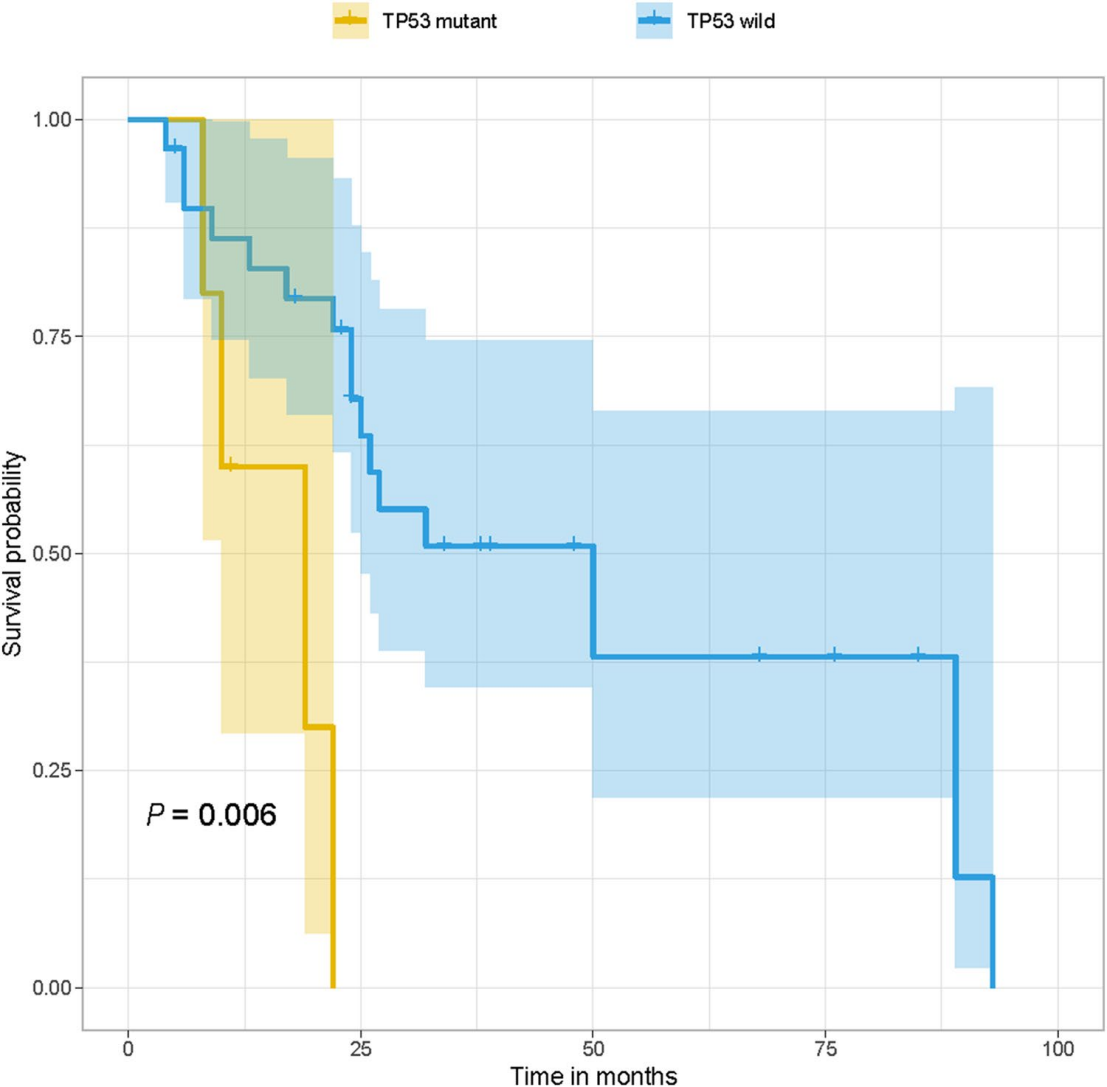
XXXX

Several pathways were disrupted in MM (Fig. 3). MAPK signaling pathway was recurrently mutated in almost all subtype of melanoma, especially highly frequently mutated in cutaneous melanoma. *BRAF* mutation occurred in 5 patients and only one mutation was the hotspot mutation V600E. 2 patients had mutations in *NRAS*. *NF1* was the most frequently mutated genes among the three major mutation type of cutaneous melanoma. As previously reported, high frequency of *KIT* mutations were detected in our MM patients. *TP53* mutated in 2 patients of anorectal melanoma and 5 patients of gynecological melanoma (Fig. 4). *ERBB2*, *FLT4* and *RPS6KA40* mutated in 2 patients. Except *KMT2D*, there were another 3 genes-*SETD1B*, *ALDH2,* and *KMT2A* of lysine degradation pathway mutated in at least 2 patients. *SETD1B* mutated in 4 patients. *KMT2A* was exclusively mutated with *KMT2D*. There were many signaling pathway that was altered in less frequency, such as cell cycle.

### *TP53* mutations associated with poor survival

The features that associated with clinical outcome were searched. Gender, tumor size and primary site were not associated with prognosis. 15.91% (7/44) of patients had mutations in *TP53*. Patients with *TP53* mutations tend to have worse clinical outcome (median survival time 19 vs. 50 months, log-rank *P* = 0.006).

## Discussion

Mucosal melanoma occurs more frequently in Asian populations than in Europeans [7]. The therapy of MM is challenging due to the underlying molecular mechanism of MM is far to be fully understood. Furthermore, it is usually detected at an advanced stage and responds to immunotherapy less frequently [28]. To explore the basic mutational profile and identify actionable/druggable biomarkers, we collected 48 MM samples from 44 Chinese patients and performed WES in this study.

We identified an epigenetic factor, *KMT2D*, was frequently mutated in our samples. According our knowledge, this was the first time to report this gene as a recurrently mutated gene in MM’s patients. We tested whether the mutation status of *KMT2D* has impact on the postoperative survival. However, the mutational status of *KMT2D* was not associated with prognosis. Other important clinical information of MM, such as the Clark level, Breslow thickness, lymph node metastasis, distant metastases, which unfortunately were not recorded for most patients in our study, need to be tested in future studies to further clarify the clinical roles of *KMT2D*. KMT2D is a histone methyltransferase that primarily performs monomethylation on the lysine 4 position of histone H3 (H3K4me1). *KMT2D* was reported to play roles in tumor progression [41, 42]. Recently, Kunal Rai1’s lab revealed that *KMT2D* was a tumor suppressor in melanoma based on an in vivo epigenome-focused pooled RNAi screen and confirmed the finding by using a genetically engineered mouse model [42]. KMT2D loss causes genome-wide reduction of H3K4me1-marked active enhancer signal which leads to repression of IGFBP5 and activated IGF1R-AKT to increase glycolysis. We also found another five genes mutated which play key roles in histone modifications. Interestingly, *KMT2A* was mutated exclusively with *KMT2D* though with less mutational frequency. Our data imply the importance of histone modification in MM.

Limited prognosis markers for MM were reported previously. *POM121* mutation was reported to have worse clinical outcomes in MM of head and neck [22]. Baseline inflammatory markers (NLR, PLR, and LMR) were revealed to be associated with clinical outcome of MM [43]. *TP53* is one of the most frequently mutated gene in human cancers. As a famous tumor suppressor, TP53 played an important role in cell proliferation and induction of cell death [44, 45]. *TP53* mutations were reported to associate with poor prognosis in breast, colorectal, head and neck, and leukemia cancer [46]. In our study, we revealed that MM with *TP53* mutations tend to have worse clinical outcome. Differences in clinical and pathological features such as primary sites and genders may influence the survival. Of all the sequenced samples, only one sample was collected from vulval region. We performed survival analysis after removing this sample. It was also shown that the survival rate was significantly different between *TP53* wild and mutation group (log-rank *P* = 0.0076). There were 3 male with survival time recorded. We performed survival analysis again after further removing the 3 male samples. TP53’s mutation still significantly influenced the survival (log-rank *P* = 0.0067). So, TP53 was related with poor prognosis independent of sex and primary site. However, the underlying mechanism of TP53 has effect on survival needs to be explored further.

The samples were collected from two locations in lower body site: anorectal region and urinary-genital tract. Generally, MM had a very low TMB as previously reported [17, 18, 21, 22, 47]. Furthermore, we found samples from anorectal locations had a significantly lower TMB than which from urinary-genital tracts (Wilcox test, *P* = 7.838e-05). It implied MM had body site-specific mutational patterns which increased the challenge of MM treatment. Though TMB was reported to be associated with prognosis in many other types of cancer, however, it was not associated with clinical outcome in our study (log-rank *P* = 0.66). Primary and metastatic tumor sites were both collected for some patients. It revealed that local recurrent and lymph node metastatic tumors had a higher or comparable TMB than tumors from primary site while urinary tract metastasis has lower TMB than primary site. However, further study is needed due to the limited samples from metastatic sites included in the study.

Our study showed that MM, as a whole population, had no dominant mutations as comparable as BRAF in cutaneous melanoma. Although some famous cancer signaling pathways, such as MAPK pathway, were still frequently mutated in MM. *KIT* and *NFI* were top mutated genes in our study which similar to previous studies on MM [18, 19, 21]. However, the mutation rates of other genes varied among different studies. For example, Jennifer and his colleagues found *SF3B1* recurrently mutated in 42.1% (8/19) in their MM samples [18]. While *SF3B1* mutated in our samples with less frequency (11%, 5/45). Moreover, *SPRED1* was not mutated in their samples. While Julien’s, Sun’s and our work all identified *SPRED1* as a recurrently mutated genes in MM [22, 24]. Julien’s team even experimentally confirmed *SPRED1* loss was a driver of mucosal melanoma using zebrafish modeling. *KMT2D* may be the same situation. It may caused by different sampling location or genetic factors. It implies high heterogeneity and complexity in MM's genome.

In this study, we revealed genes in MAPK signaling pathway, such as *KIT* and *NF1*, were top recurrently mutated genes in MM as previous studies. Besides, another 18 genes, such as *ERBB2*, *FLT4*, *RPS6KA4*, were also identified mutated in MM patients, which expand the patients benefit from MAPK inhibitor. Interestingly, we revealed genes in histone modifications were recurrently mutated in MM patient. Among which, *KMT2D*, was firstly identified as top mutated genes in MM patient. We revealed that MM with *TP53* mutations tend to have short postoperative survival time which is independent of gender and sampling location. However, there are several limitations in our studies. First, the clinical roles of *KMT2D* mutations remain unclear due to the important clinical information, such as Clark level, Breslow thickness, lymph node metastasis, distant metastases, for most patient were not recorded. Second, the underlying mechanism that TP53 has impact on patient’s survival needs to be explored further. Third, due to high heterogeneity and complexity of MM, more samples are needed in future study to reveal the whole mutational profile of MM.

### Supplementary Information

Below is the link to the electronic supplementary material.

Supplementary file1 (DOCX 477 KB)

Supplementary file2 (XLSX 13 KB)

Supplementary file3 (XLSX 20 KB)

## Data Availability

The corresponding authors are pleased to provide the original data to those who are interested.
