# Supplementary material for: Whole-exome sequencing reveals mutational profiles of anorectal and gynecological melanoma
Source: Med Oncol. 2023 Oct 13;40(11):330. doi: 10.1007/s12032-023-02192-6 (PMC10575813; doi:10.1007/s12032-023-02192-6)
Supplement: Supplementary file 1 — Supplementary file1 (DOCX 477 KB) [file 12032_2023_2192_MOESM1_ESM.docx]

**Supplementary material online**

**Figure S1.** TMB is not associated with clinical outcome.

**
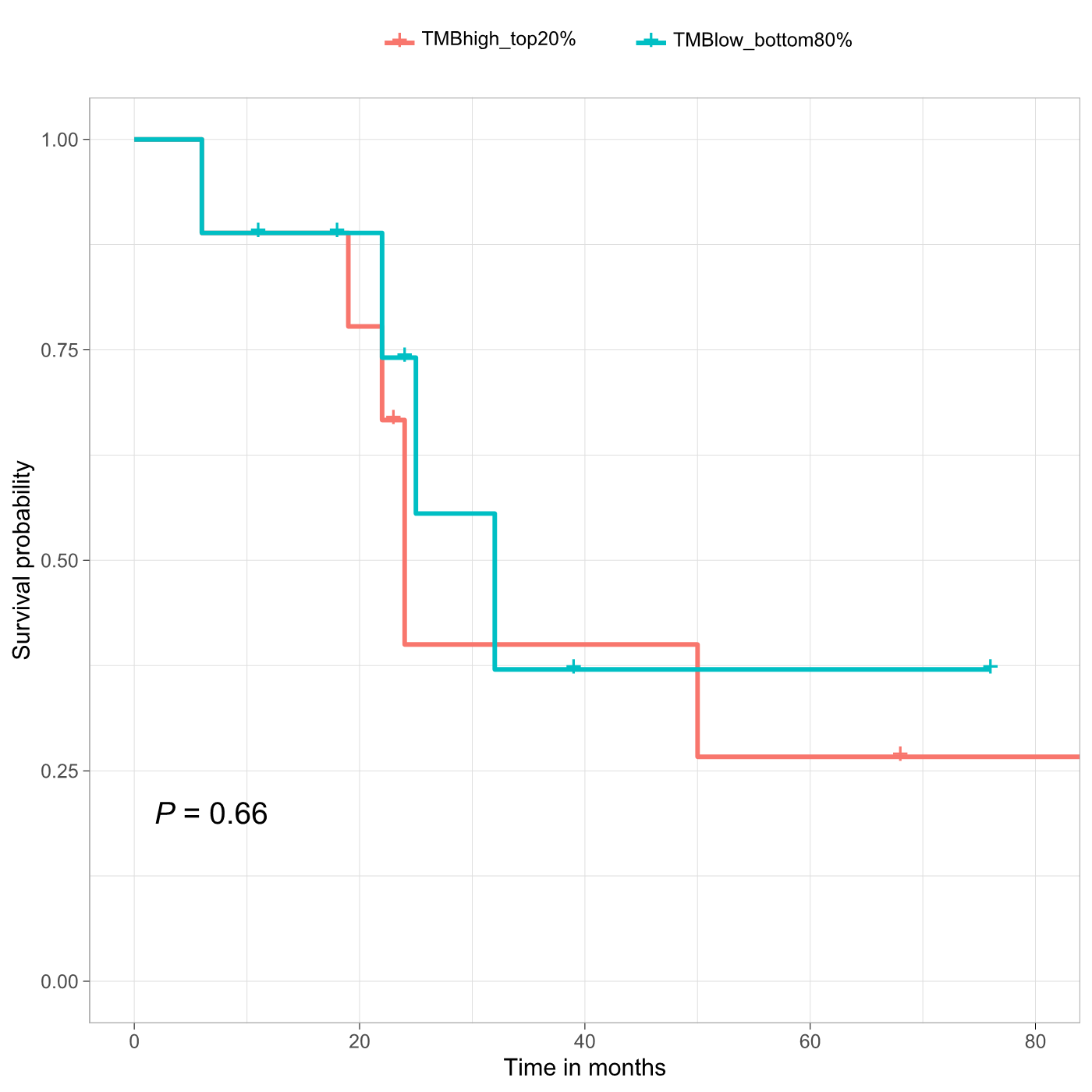
**


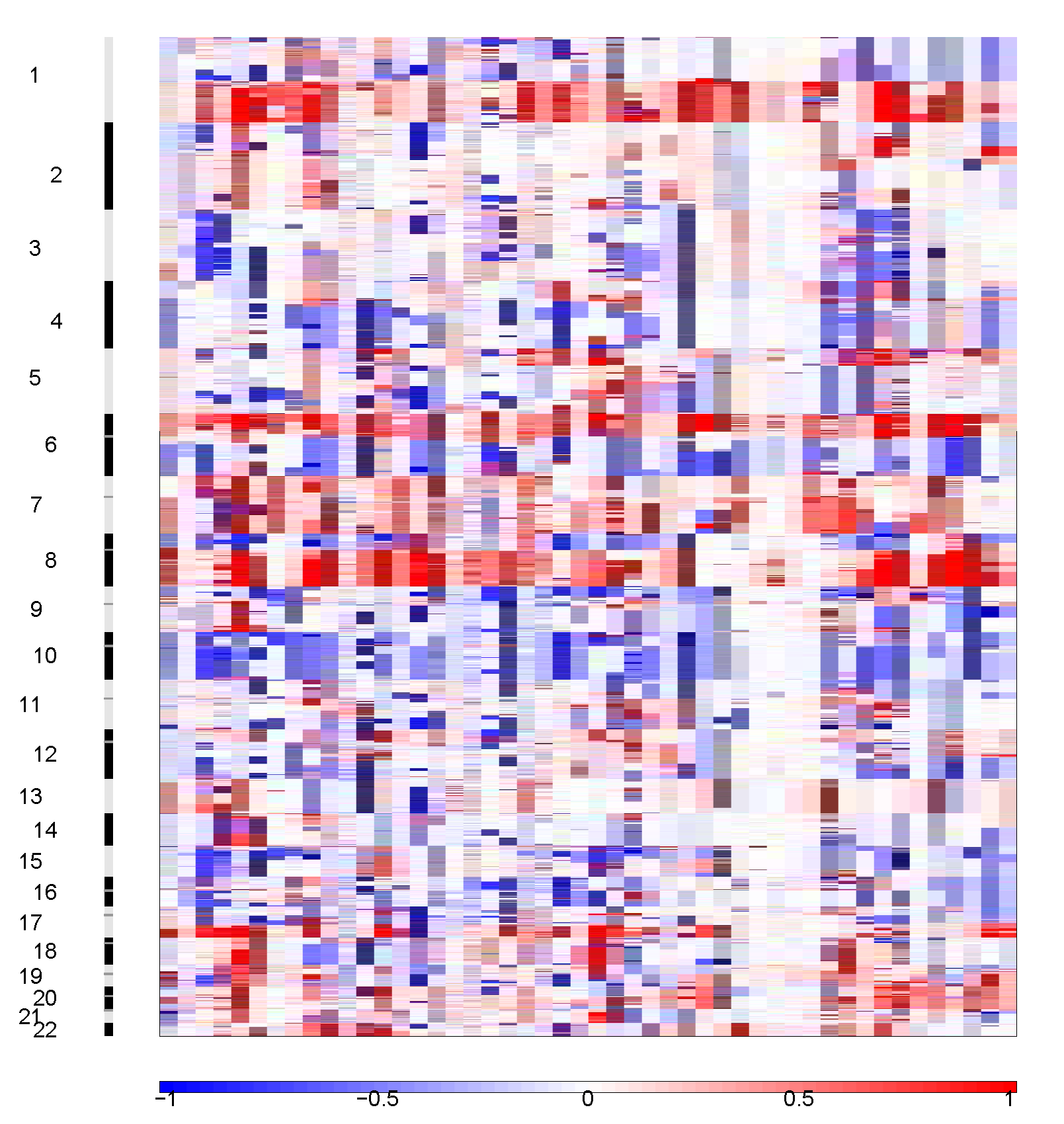
**Figure S2.** Heatmap of copy numbers in all samples. Color indicates the log2 ratio of read depth from low (blue) to high (red).
